# Neural substrates of social facilitation effects on incentive-based performance

**DOI:** 10.1093/scan/nsy024

**Published:** 2018-04-10

**Authors:** Vikram S Chib, Ryo Adachi, John P O’Doherty

**Affiliations:** 1 Department of Biomedical Engineering, Johns Hopkins School of Medicine, Baltimore, MD 21205, USA; 2 Kennedy Krieger Institute, Baltimore, MD, USA; 3 Division of Biology and Biological Engineering; 4 Division of Humanities and Social Sciences; 5 Computation and Neural Systems, California Institute of Technology, Pasadena, CA 91125, USA

**Keywords:** social facilitation, reward, motor, prefrontal cortex, ventral striatum

## Abstract

Throughout our lives we must perform tasks while being observed by others. Previous studies have shown that the presence of an audience can cause increases in an individual’s performance as compared to when they are not being observed—a phenomenon called ‘social facilitation’. However, the neural mechanisms underlying this effect, in the context of skilled-task performance for monetary incentives, are not well understood. We used functional magnetic resonance imaging to monitor brain activity while healthy human participants performed a skilled-task during conditions in which they were paid based on their performance and observed and not observed by an audience. We found that during social facilitation, social signals represented in the dorsomedial prefrontal cortex (dmPFC) enhanced reward value computations in ventromedial prefrontal cortex (vmPFC). We also found that functional connectivity between dmPFC and ventral striatum was enhanced when participants exhibited social facilitation effects, indicative of a means by which social signals serve to modulate brain regions involved in regulating behavioral motivation. These findings illustrate how neural processing of social judgments gives rise to the enhanced motivational state that results in social facilitation of incentive-based performance.

## Introduction

A number of studies have reported that an individual’s performance accuracy or level of effort exertion can be improved when working in the presence of others. These behavioral effects have been shown in a diversity of species [e.g., humans (Bond and Titus, 1983; Baumeister, 1984; Strauss, 2002), monkeys (Harlow and Yudin, 1933; Munkenbeck Fragaszy and Visalberghi, 1990; Visalberghi and Addessi, 2001), insects (Zajonc and Herman, 1969; Miramontes and DeSouza, 1996)] and can take the form of audience effects in which the mere presence of spectators causes increases in performance. Previous psychological explanations have focused on the effects of increased arousal (Zajonc, 1965, 1980), changes in attention (Sanders, 1981; Bond, 1982; Baron, 1986), or worries about self presentation (Bond, 1982; Bond and Titus, 1983) arising from observations by an audience.

Recently, human neuroimaging studies have begun to examine how social facilitation effects are processed in the brain. These works have studied how audience effects influence charitable donation (Izuma *et al.*, 2010), unskilled physical exertion (Yoshie *et al.*, 2016) and cognitive tasks (Müller-Pinzler *et al.*, 2015; Dumontheil *et al.*, 2016), and have shown that when subjects were observed they exhibited increased activity in brain networks related to mentalizing (i.e. medial prefrontal cortex, parietal cortex and intraparietal cortex). Indeed, dorsal aspects of medial prefrontal cortex show enhanced activations when observed by an audience, even when not performing a task or being evaluated (Somerville *et al.*, 2013). Notably, these studies did not attempt to dissociate neural processes related to audience observation and motivated performance, and none of these studies examined how skilled-performance for monetary incentives, and associated reward representations, were influenced by the presence of an audience.

Building on a large literature demonstrating a strong association (whether facilitatory or deleterious) between incentives and behavioral performance (Ariely *et al.*, 2009; Mobbs *et al.*, 2009; Chib *et al.*, 2012; Chib *et al.*, 2014), as well as a number of studies that have implicated brain regions involved in encoding reward valuation also being involved in encoding of social valuations (Behrens *et al.*, 2008; Lin *et al.*, 2012; Janowski *et al.*, 2013; Ruff and Fehr, 2014), we propose an incentive-based account of social facilitation effects during performance for monetary incentives. We suggest that successful incentivized performance in the presence of an audience is evaluated as a positive outcome, because performing well in front of others is perceived as increasing social approval by others. Conversely, performing poorly in the presence of an audience results in a perceived adverse impact on one’s social standing. As a result, we hypothesize that individuals will be more motivated to perform a task successfully in front of an audience compared to a scenario in which no audience is present. In essence, the audience serves to increase the incentive motivation for successful performance. To test this hypothesis, we measured behavior and collected functional magnetic resonance imaging (fMRI) data while participants performed an extensively trained, incentivized, skilled motor task during conditions in which their performance was observed by an audience and conditions in which they were not observed.

Our incentive-based hypothesis of social facilitation makes a number of distinct predictions about neural responses. First, activity in brain regions sensitive to outcome value such as the ventromedial prefrontal cortex (vmPFC) (Rangel *et al.*, 2008; Chib *et al.*, 2009; Fitzgerald *et al.*, 2009; Levy and Glimcher, 2012), will show enhanced activity in response to success compared to failure in observed relative to unobserved trials during a monetarily incentivized task. Second, during trials in which an audience observes a participant’s performance, circuits involved in representing the thoughts and intentions of other individuals, including the dorsomedial prefrontal cortex (dmPFC), temporal parietal junction (TPJ) or posterior superior temporal sulcus (STS) will be engaged (Siegal and Varley, 2002; Frith and Frith, 2006; Saxe, 2006; Somerville *et al.*, 2013; Müller-Pinzler *et al.*, 2015; Dumontheil *et al.*, 2016; Yoshie *et al.*, 2016). Third, considering a number of studies showing evidence that dmPFC plays a central role in the ability to make inferences about the mental states of others in general and during social value-based decision-making (Siegal and Varley, 2002; Amodio and Frith, 2006; Hampton *et al.*, 2008;De Martino *et al.*, 2013), we hypothesized there will be increased functional connectivity between dmPFC and outcome value areas (vmPFC) in the observed *vs* unobserved conditions. This interaction will reflect the degree to which awareness of being monitored modulates the reward-related responses to success and failure. Fourth, since being observed will result in enhanced motivation to perform successfully, neural responses will be enhanced in the ventral striatum (vSTR), a key brain structure that us and others have previously implicated in motivated instrumental motor performance (Bray *et al.*, 2008; Talmi *et al.*, 2008; Balleine and O’Doherty, 2010; Chib *et al.*, 2012, 2014;).

## Materials and methods

### Experimental design

Stimulus presentation and behavioral data acquisition were implemented using custom designed Matlab (http://www.mathworks.com) and C++ programs implementing the OpenGL (Silicon Graphics Inc., USA) graphics libraries. During fMRI, visual feedback of targets and hand position were presented via a projector positioned at the back of the scanning room. Participants viewed a reflection of the projector image (800×600 pixels) in a mirror attached to the scanner head coil. This system allowed us to display virtual images, video of observers, and manipulate visual feedback.

Direct view of participants’ arms was obscured since they were positioned in the scanner head-first-supine and the display mirror blocked their view. A Vicon motion tracking system (MX Ultranet system, with 4 MX40+ cameras; Oxford Metrics Ltd., Oxford, UK) was used to record the motion of an infrared reflective maker attached to the right index finger. During experiments, these signals were sent to our custom designed software for real-time visual feedback of participants’ hand position. The position signals were also recorded for further offline analysis. Participants’ arm movements were confined to the coronal plane, and visual feedback of these movements was presented in 2 D on the visual display.

### Experimental setup

#### Participants

All participants were right handed, and were prescreened to exclude those with a prior history of neurological or psychiatric illness. The California Institute of Technology Institutional Review Board approved this study, and all participants gave informed consent.

Twenty participants (mean age, 24.5; age range 19–32; 7 females) took part in the motor experiment. Forty male participants, in pairs of two, served as observers of the participants performing the motor experiment. Male participants were always used as observers to remove any experimental confounds that could be present related to gender. Scanned participants and observers did not know each other before performing the experiment, and participants were recruited from the Caltech student population and the greater Pasadena/Los Angeles community. Participants performing the motor experiment had never previously performed the motor task. Of the 20 participants performing the motor experiment, 1 participant was excluded from the MRI analysis because of excessive movement artifact, another participant was excluded from the social anxiety correlation because she did not complete the Liebowetz Social Anxiety Scale.

#### Motor task

We modified an experimental task that we have used in the past to examine skilled-task performance under pressure (Chib *et al.*, 2012, 2014). The experiment was comprised of three phases that took place on two consecutive days. On the first day participants practiced control of the spring-mass system (training phase). For a more detailed description of the spring-mass system see Chib *et al.* (2012). After the training phase we determined participants’ rates of success at various target sizes (thresholding phase). On the second day participants controlled the spring-mass system with the purpose of winning money in the context of being watched or not being watched (testing phase). Both the training and thresholding phases took place in a mock scanner to replicate the posture necessary in the scanning environment. The testing phase took place in the fMRI scanner. Prior to the experiment participants were told they would receive a show-up fee of $40 dollars at the end of experiment.

The training phase was comprised of 500 trials. A trial began when a participant put her hand cursor over the start position (x) and ended after 2 s. At the end of the trial, the cursors flashed green if the scoring criteria were met and red otherwise. The target size was 50 mm^2^ throughout the training phase. The thresholding phase was the same as the training phase in all respects, except that it was comprised of 200 trials of varying size. Target sizes range from 10  to 55 mm^2^ in increments of 5 mm^2^. Each target size was randomly presented 20 times. From this data we obtained a psychometric curve that represented participants’ performance over a range of target sizes.

During the testing phase participants were scanned with fMRI while controlling the spring-mass system for reward. During this phase 2 observers were seated in the scanning control room and participants were able to see a live video stream of the observers on given trials during the motor experiment. On select trials, the observers viewed a monitor displaying the motor performance of participants. Before beginning the testing phase participants were introduced to the observers so that they knew the observers were actually present and watching the experiment. On trials in which participants were watched, the observers recorded on a score sheet if a participant was successful or unsuccessful in performing the task. Prior to the experiment, participants were told about the scoring by observers and that that they would be shown their score sheet at the end of the experiment. Together, these conditions ensured that participants were cognizant of the fact that they were being watched and evaluated during the observation conditions.

Participants were told that at the end of the experiment one trial would be randomly selected and a payment made based on their performance on that trial. This payout mechanism ensured that participants evaluated each trial independently. Participants performed all trials for $25 and under conditions of observation or no observation. The conditions consisted of trials in which participants were observed and saw a live video stream of the observers watching them, observed and saw a scrambled video of the observers, were not observed and saw a prerecorded video of observers not present during the experiment and were not observed and saw a prerecorded scrambled video. Using psychometric curves generated during the thresholding phase, a target size was created for each participant such that it coincided with a 60% unincentivized success rate. Each experimental condition was randomly presented 40 times for a total of 160 trials. At the beginning of each trial, participants were shown a message indicating the amount of incentive they were playing for and the experimental condition (jittered duration 2–5 s). They then performed the motor task, with the same success criteria as during training (duration 2 s), and were shown the trial outcome (1 s). At the end of each participants’ testing phase a single trial from their phase was selected at random and the participant was paid based on performance on that trial.

To summarize, our task has several important features: (1) since our aim was to examine how performance in front of an audience serves to enhance incentive based motivation and social facilitation, presentation of the incentive cues (i.e. reward value and audience condition) and task execution were dissociated in time. This enabled us to separately examine signals related to evaluation of the audience/incentive conditions and processes related to task execution and outcome value. Notably, previous studies of audience effects were not designed to disentangle signals related to processing of audience observation, motivational performance and outcome reward value (Morishima *et al.*, 2012; Müller-Pinzler *et al.*, 2015; Dumontheil *et al.*, 2016). (2) Since participants were highly trained before entering the main experimental condition, early in motor performance they had a strong sense about their potential outcome on a given trial (i.e. success/failure), even before they were presented with the final outcome. In our previous studies of value-based motor performance we consistently reported decreases in outcome value encoding on unsuccessful trials compared to successful trials (Chib *et al.* 2012, 2014), and we leveraged this finding to examine how outcome values were modulated by audience observation during task performance.

The original goal of our project was to investigate performance deterioration effects (i.e. choking under pressure) of social observation as opposed to performance facilitation effects. However, preliminary behavioral experiments conducted before the acquisition of the fMRI experiment reported here, revealed a performance facilitation effect in the range of incentives from $0–$50 instead of a choking effect. As a consequence, we implemented the current fMRI experiment to examine the neural mechanisms underlying this social facilitation effect, using a fixed incentive level of $25 which was within the range of incentive levels we previously found to show facilitation effects.

#### MRI protocol

A 3-Tesla Siemens Trio (Erlangen, Germany) scanner and standard radio frequency coil was used for all the MR scanning sessions. To reduce the possibility of head movement related artifact, participants’ heads were securely positioned with foam position pillows. High resolution structural images were collected using a standard MPRAGE pulse sequence, providing full brain coverage at a resolution of 1 mm×1 mm×1 mm. Functional images were collected at an angle of 30° degrees from the anterior commisure–posterior commisure (AC–PC) axis, which reduced signal dropout in the reward-related brain areas relative to AC–PC aligned images (Deichmann *et al.*, 2003). Forty-five slices were acquired at a resolution of 3 mm×3 mm×3 mm, providing whole-brain coverage. A one-shot echo-planar imaging (EPI) pulse sequence was used [TR = 2800 ms, TE = 30 ms, FOV = 100 mm, flip angle = 80°].

#### Self-report questionnaires

To assess the extent of participants’ anxiety associated with social interaction and performance situations we implemented the Liebowitz Social Anxiety Scale (LSAS) (Heimberg *et al.*, 1999). This scale is made of two sub scales that assess fear and avoidance of social situations. Combining these sub scales returns a metric between 0 and 144 points that is widely used to characterize the level of an individual’s general propensity to exhibit social anxiety.

Participants also performed the Autism Spectrum Quotient questionnaire (Woodbury-Smith *et al.*, 2005) and the Behavioral Avoidance/Inhibition Scales (Carver and White, 1994); however, these measures were not analyzed further in the context of this study.

### Data analysis

#### Behavioral performance analysis

We evaluated participants’ behavioral performance as their percent success in the different experimental conditions. We focused on comparing behavioral performance between the observed and unobserved conditions.

#### Kinematic trajectory analysis

To ensure participants’ movement kinematics were the same across the observed and unobserved conditions and that our imaging results were not confounded by differing motor output for these conditions, we calculated measures of hand kinematics during successful task performance.

##### Hand accuracy

Endpoint accuracy was calculated as the distance between a participant’s final hand position and the target center (Euclidean norm). We found no differences in the hand accuracy metric between the observed and unobserved conditions (*t*_19_* *= 0.82, *P *= 0.42).

##### Mean velocity

This velocity metric was the mean hand velocity over the course of a movement trajectory. We found no differences in the velocity metric between the observed and unobserved conditions (*t*_19_* *= 0.26, *P *= 0.80).

##### Hand smoothness

This metric captures the average rate of change of the acceleration of movement (jerk). It is calculated by dividing the negative mean jerk magnitude by the peak speed. Taking the negative of the mean jerk causes increases in the jerk metric to correspond with increases in smoothness; in this way it transforms the jerk metric from a measure of ‘non-smoothness’ into a measure of smoothness. Normalizing the mean jerk by the peak speed makes the measure robust to confounds arising from changes in overall movement speed. This method was introduced in Roher *et al.* (2002). We found no differences in the hand smoothness metric between the observed and unobserved conditions (*t*_19_* *= 1.19, *P *= 0.25).

#### Image analysis

The SPM8 software package was used to analyze the fMRI data (Wellcome Department of Imaging Neuroscience, Institute of Neurology, London, UK). A slice-timing correction was applied to the functional images to adjust for the fact that different slices within each image were acquired at slightly different points in time. Images were corrected for participant motion, spatially transformed to match a standard EPI template brain included in the SPM software package, and smoothed using a three-dimensional Gaussian kernel (6 mm FWHM) to account for anatomical differences between participants. This set of data was then analyzed statistically.

#### General linear model

A general linear model (GLM) was used to generate voxel-wise statistical parametric maps (SPMs) from the fMRI data. We created participant-specific GLMs that modeled the time of condition presentation and the time of the motor task. We modeled two types of condition presentation: trials in which participants were observed and not observed. We included scrambled video conditions to control for possible visual confounds in any potential post-hoc imaging analyses. However for all analyses presented here, we collapsed across scrambled and unscrambled video conditions to increase the statistical power of our observed and unobserved conditions. For the motor task, we modeled trials in which participants were observed and successfully performed the task, were observed and unsuccessful, were not observed and successful, and were not observed and unsuccessful. This resulted in a grand total of six modeled conditions. The incentive presentation events were modeled with a duration lasting the length of incentive presentation (2–5 s), while the motor task events were modeled with a fixed duration of 2 s. In addition, regressors modeling the head motion as derived from the affine part of the realignment produce were included in the model.

With this model we tested brain areas in which activity was related to being observed at the time of incentive presentation and during the motor task. This was done by creating contrasts with the aforementioned conditions at the times of incentive presentation and the motor task.

#### Experimental design and statistical analysis

Nineteen participants were included in the analysis of the fMRI and behavioral data. All participants performed all experimental conditions: observed by an audience with a live video feed of the audience; observed by an audience with a live scrambled video feed of the audience; not observed by an audience with a prerecorded video; not observed by an audience with a scrambled prerecorded video. We recorded participants performance (success/failure and trajectory kinematics) on these trials. To assess social facilitation effects we collapsed across the video conditions and performed a paired *t* test between the observed and the unobserved conditions.

Second-level group contrasts from our GLM were calculated as a one-sample *t* test against zero for each first-level linear contrast. All activations were reported as significant if they survived family-wise error correction for multiple comparisons across a sphere with radius 8 mm (SVC) centered on the peak of activity isolated in independent studies. Illustrative bar plots, illustrative covariate plots and our ROI analysis were generated using coordinates centered on an 8 mm sphere at the peak of activity isolated in independent studies. For vmPFC, we used the coordinates [0, 53, 4] taken from Suzuki *et al.* (2012) which reported decision-value signals in this region during neuroeconomic choice in a social context; for dmPFC, we used the coordinates [3, 51, 24] taken from Hampton *et al.* (2008) which reported activity related to social context and functional connectivity between this region and vmPFC during social value-based decision-making.; for rTPJ and lTPJ, we used the coordinates [51, −54, 27] and [−54, 60, 21] taken from Saxe and Kanwisher (2003) which showed these regions to be related the theory of mind; for premotor cortex, we used the coordinates [−30, −4, 61] taken from Chib *et al.* (2014) which used this exact same motor task as in this study. For vSTR we used an a priori anatomically defined ROI (encompassing nucleus accumbens and ventral parts of the putamen). This exact ROI was used in our previous studies examining skilled performance for incentives (Chib *et al.*, 2012, 2014). All ROIs were defined a priori and specified in Montreal Neurological Institute stereotactic space coordinates.

The plots shown in Figures 2G and 3B, D and F are for illustrative purposes, in order to clarify the activation patterns in their associated contrasts. For completeness, we also conducted whole-brain analyses to complement our ROI analyses, and we report the results of those whole-brain analyses in the tables. Statistical inference was carried out in the SPM framework.

#### Psychophysiological interaction (PPI) analysis

To assess changes in brain region connectivity as a function of task performance and being watched by others, we carried out a PPI analysis. PPI is a measure of context-dependent connectivity, which explains the regional activity of other brain regions in terms of the interaction between responses in a seed region and cognitive or sensory processes. We estimated the following PPI models using the generalized PPI toolbox for SPM (McLaren *et al.*, 2012):

PPI 1: The goal of this analysis was to investigate if activity in the dmPFC was correlated with activity in the vmPFC at the time of task execution as a function of social facilitation.

For this analysis the PPI term was defined as *Y* × *P*. The physiological variable *Y* was the average blood oxygen level-dependent (BOLD) time course taken from all voxels in 6 mm spheres surrounding the peak coordinates in the dmPFC seed [*x* = −3; *y* = 53; *z* = 25]. These peak coordinates for dmPFC were obtained from the contrast reported in Figure 2C, restricting the contrast to the independent ROI definition (an 8 mm sphere). The psychological variable *P* was the trial by trial interaction between a participant’s behavioral performance (successful/unsuccessful) and observation conditions (observed/unobserved). This PPI was calculated for events occurring at the time of task performance.

We entered the physiological variables *Y*, the psychological variables *P*, the PPI interaction terms and movement regressors into a new GLM. Importantly, this GLM also contained the main experimental conditions. Thus, any effects on the PPI interaction reveal coupling that could not be explained from the mutual correlation of the seed and target regions with performance or observation factors. The PPI contrast illustrates areas in which coupling between dmPFC is enhanced or decreased as a function of task performance (success/failure) and observation (observed/unobserved) conditions.

PPI 2. The goal of this analysis was to investigate if activity in the vSTR was correlated with activity in the premotor cortex at the time of task execution as a function of social facilitation.

This analysis was identical to the previous PPI. The physiological variable *Y* was the BOLD time course taken from all voxels in two 6 mm spheres surrounding the peak coordinates in the right [*x* = 15; *y* = 11; *z* = −8] and left vSTR seeds [*x* = −15; *y* = 5; *z* = −11]. These peak coordinates for vSTR were obtained from the contrast reported in Figure 3A. The psychological variable *P* was the trial by trial interaction between a participant’s behavioral performance (successful/unsuccessful) and observation conditions (observed/unobserved). This PPI was calculated for events occurring at the time of task performance.

We entered the physiological variables *Y*, the psychological variables *P*, the PPI interaction terms and movement regressors into a new GLM. The PPI contrast illustrates areas in which coupling between vSTR is enhanced or decreased as a function of task performance (success/failure) and observation (observed/unobserved) conditions.

PPI 3. The goal of this analysis was to investigate if activity in the vSTR was correlated with activity in dmPFC at the time of condition presentation as a function of social facilitation during subsequent task execution.

This analysis was identical to the previous PPI. The physiological variable *Y* was the BOLD time course taken from all voxels in two 6 mm spheres surrounding the peak coordinates in right [*x* = 15; *y* = 11; *z* = −8] and left vSTR seeds [*x* = −15; *y* = 5; *z* = −11]. These peak coordinates for vSTR were obtained from the contrast reported in Figure 3A. The psychological variable *P* was the trial-by-trial interaction between a participant’s behavioral performance (successful/unsuccessful) and observation conditions (observed/unobserved). A critical difference between this PPI and the previous PPIs was that the psychological regressors in this PPI were extracted for events at the time of condition presentation (i.e. before the task execution). This allowed us to test how functional coupling before task execution was related to subsequent social facilitation effects.

We entered the physiological variables *Y*, the psychological variables *P*, the PPI interaction terms and movement regressors into a new GLM. The PPI contrast illustrates areas in which coupling between vSTR is enhanced or decreased as a function of task performance (success/failure) and observation (observed/unobserved) conditions.

## Results

**Fig. 1.**
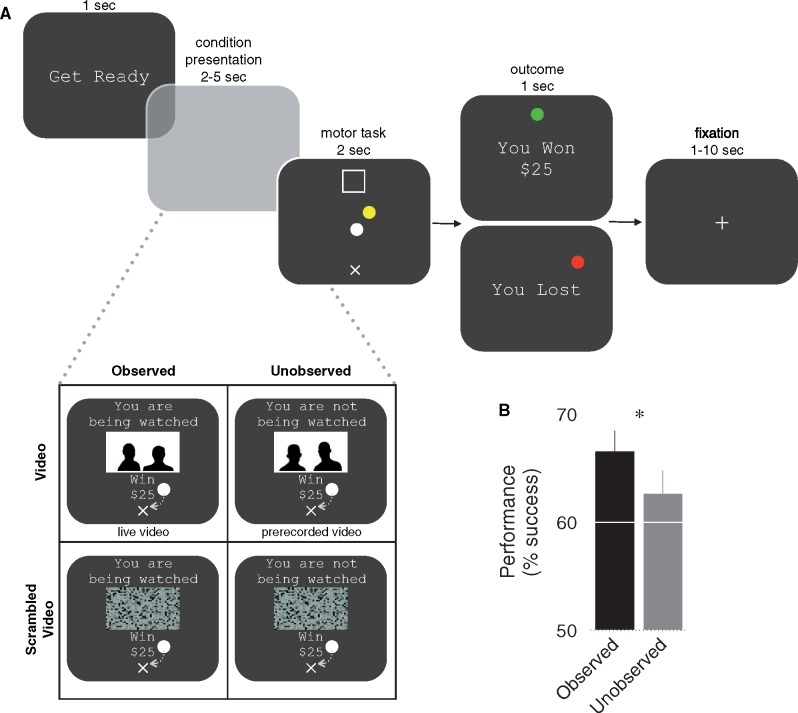
Experimental task and behavioral performance. (A) At the beginning of each trial participants were informed about which experimental condition they were in. Trials were always for $25. There were two observation conditions: one in which participants saw a live video stream of people in another room who were evaluating their task performance (observed condition); and another condition in which participants saw a prerecorded video of people who were unfamiliar to them and not evaluating their performance (unobserved condition). These conditions had a counterpart scrambled video condition. During condition presentation, to initiate the motor task, participants placed their white hand cursor in the start position (x) for a random amount of time (2–5 s). During the task, a target (□) appeared that was registered to a position 20 cm distal from the start. To successfully perform the task, participants had to place their hand cursor and a mass cursor into the target within 2 s, while achieving a final velocity below 0.02 m/s. At the end of the trial they were shown a message indicating the outcome of their performance. In the case that a participant successfully placed the spring-mass in the target, a positive message was displayed (‘You Won $25’); otherwise, the participant was informed of her negative outcome (‘You Lost’). (B) Participants’ performance was significantly improved on trials in which their performance was observed by others (*P < 0.05). Error bars represent SEM.

Behavioral results indicate that participants’ average level of success was significantly improved in the observed condition as compared to the unobserved condition, illustrating the social facilitation effect of performance (*t*_19_* *= 2.54, *P *= 0.02) (Figure 1B).

### Neural representations of performance outcome value

**Fig. 2.**
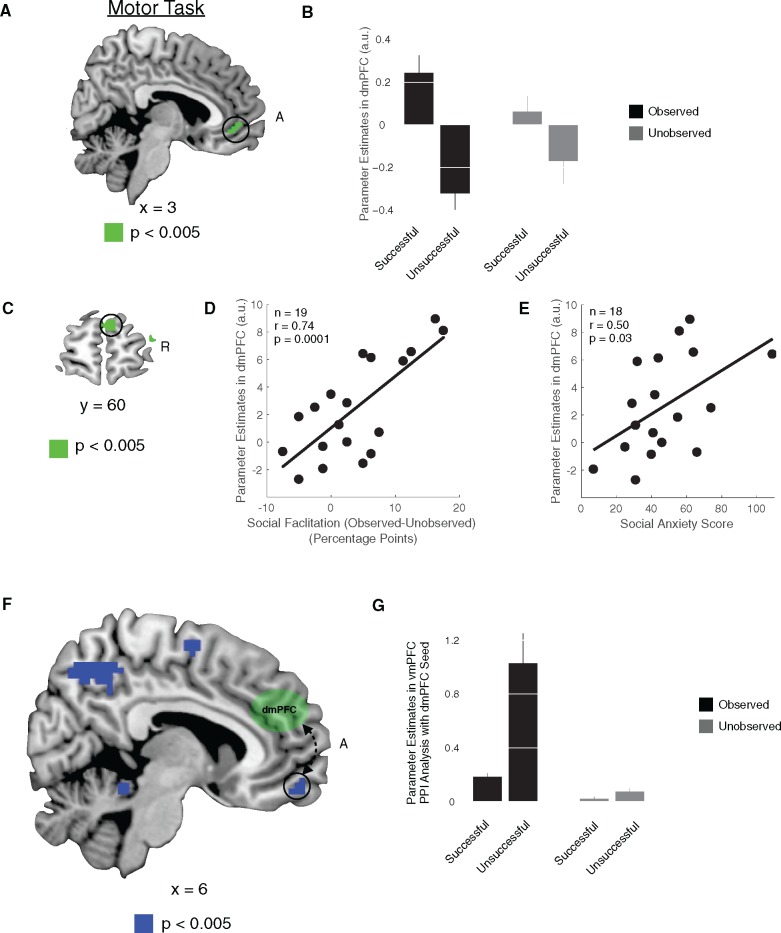
Neural representations of value and social facilitation. (A) At the time of task execution, vmPFC exhibited significant increases in signal when comparing conditions in which participants’ performance was successful and unsuccessful. (B) A significant interaction in vmPFC was found between observation conditions and performance. (C) Activity in dmPFC was positively modulated by an individual’s propensity for social facilitation. Between-participant regression analysis considering the difference in performance between the observed and unobserved conditions as a covariate for observation-related activity in dmPFC (i.e. difference between the observed and unobserved imaging conditions) (D) Plot of the correlation between activity in the dmPFC and social facilitation of performance (i.e. illustration of Figure 2C). (E) Plot of the correlation between activity in the dmPFC and social anxiety scores. (F) A connectivity analysis with dmPFC seed found that the functional coupling between dmPFC and vmPFC was significantly increased during trials in which participants were observed and they were unsuccessful. (G) Bar plot of the vmPFC PPI effects for the observed/unobserved conditions and successful/unsuccessful performance. All contrasts are significant at *P* < 0.05, small volume corrected. Error bars denote SEM. R, right. A, anterior.

We began testing our neural predictions of incentive-based social facilitation by estimating a GLM of BOLD activity that included regressors for the different observation conditions at the time of incentive presentation; and for the different observation conditions, separated by success or failure, at the time of the motor task. To confirm an effect of performance outcome value in vmPFC, irrespective of observation, we compared activity in vmPFC between trials in which individuals were successful and unsuccessful during motor task performance, pooling across observation conditions. It is important to note that since the task required implementing rapid skilled motor response toward a target, over a 2-s interval, which was followed immediately (without jitter) by explicit feedback (informing participants about winning or losing), we pooled over the trial components when participants performed the motor action and received feedback. This is justified because the hemodynamic responses of these trial components are not dissociable due to the rapid presentation and sluggishness of the BOLD response; and participants could easily determine whether task performance on a given trial was a success or failure depending on their motor performance even before they received the explicit feedback. These features allowed us to observe modulation of neural activity related success or failure, and associated outcome values, when testing for effects during the motor performance phase. In accordance with higher reward outcome values for trials that were successful, as compared to those that were unsuccessful, we found increased activity in vmPFC on successful trials compared to unsuccessful trials (Figure 2A, Table 1). This result replicates our previous findings in non-social reward-based motor performance, in which participants performed the exact same motor task and we observed outcome value encoding at the time of execution related to successful/unsuccessful performance (Chib *et al.*, 2012, 2014). To examine our first prediction, that reward outcome value representations would be increased as a function of observation, we tested for an interaction between performance outcome and observation trials. An ROI analysis of vmPFC found a significant interaction effect in vmPFC consistent with enhanced reward outcome encoding during observed trials that were successful *vs* unsuccessful compared to unobserved trials that were successful *vs* unsuccessful [Figure 2B; vmPFC ROI Analysis: *F*(1, 18) = 4.42, *P *= 0.04]. These results indicate that the neural representation of value is increased on trials in which participants are observed. To further test our prediction, and control for differences in kinematic performance between successful and unsuccessful trials, we also examined activity during successful and unsuccessful trials separately because these trials exhibited no kinematic differences across observation conditions. This analysis confirmed that vmPFC was more active during the observed condition compared to the unobserved condition [vmPFC ROI Analysis: successful trials *t*(18)= 3.34, *P* = 0.004; unsuccessful trials *t*(18)= 2.41, *P* = 0.03].

**Table 1.** Regions with a significant increase in fMRI signal for successful *vs* unsuccessful conditions at the time of task execution (Figure 2A) (*P *<0.005 uncorrected)

Region	Laterality	*x*	*y*	*z*	*T*-value
Ventromedial prefrontal cortex	C	3	44	−5	4.25
Ventral striatum	L	−12	2	−14	7.62
Ventral striatum	R	12	5	−14	5.85
Occipital cortex	R	−27	−85	−8	4.49
Occipital cortex	L	30	−88	−2	4.85
Parietal cortex	L	−42	−34	37	3.84

### Testing for the effects of imbalanced numbers of successful *vs* unsuccessful trials

Social facilitation of performance results in a greater number of successful trials in the observed condition than the unobserved condition. To ensure our imaging results comparing observed and unobserved conditions were not an artifact of an imbalanced number of trials, we randomly resampled participants fMRI data to ensure the number of trials were balanced between conditions, and performed first and second level imaging analyses on this resampled data. We began by determining, for each participant, the minimum number of trials *t*_min_ in the successful observed and unobserved conditions. We then selected, randomly, without replacement, *t*_min_ trials from each condition to ensure that the successful observed and unobserved conditions contained the same number of trials for each participant. We used a GLM to generate voxel-wise SPMs for the fMRI data for each participant. Excess trials that were not selected during the random extraction were modeled as a separate condition in our GLM. Using these SPMs we created single participant contrasts comparing successful observed and unobserved trials. These contrasts were used to create a second-level group contrast for each fMRI experiment simulation. We repeated this procedure 10 times to ensure that these results were invariant as to the specific trials chosen, and we extracted parameter estimates in ROIs in vmPFC and tested whether activity in this region was significantly increased when comparing the observed and unobserved conditions in which these conditions had an equal number of trials. In 10 out of 10 simulations, activity was significantly increased (*P* < 0.05), suggesting that our imaging results were not an artifact of an imbalance in the number of trials between the observed and unobserved conditions.

### Neural representations of social facilitation

Our second prediction was that during the social observation condition, brain regions involved in social cognition and in particular those areas involved in representing the thoughts and intentions of others, would be engaged (Siegal and Varley, 2002; Frith and Frith, 2006; Saxe, 2006). This hypothesis is supported by supplementary whole-brain analyses identifying that dmPFC and bilateral TPJ showed increased activity on social observation trials compared to non-observation trials that were successful (Table 2).

**Table 2.** Regions with a significant increase in fMRI signal for observed *vs* unobserved conditions at the time of task execution (*P *<0.005 uncorrected)

Region	Laterality	*x*	*y*	*z*	*T*-value
Ventromedial prefrontal cortex	C	15	53	−5	4.32
Dorsomedial prefrontal cortex	C	6	62	25	4.23
Temporal parietal junction	R	54	−46	19	4.46
Temporal parietal junction	L	−54	−46	40	3.8
Precuneus	C	9	−58	43	6.26

For further analyses, we focused on the dmPFC, because of a body of work suggesting that this region of the prefrontal cortex plays a role in the ability to make inferences about the mental states of others, in general, and during social value-based decision-making (Siegal and Varley, 2002; Amodio and Frith, 2006; Hampton *et al.*, 2008; De Martino *et al.*, 2013). Notably, previous neuroimaging studies of audience effects have also reported increased activation in dorsal aspects of mPFC (Somerville *et al.*, 2013; Müller-Pinzler *et al.*, 2015). If activity isolated in dmPFC during observed trials reflects social cognition related to the process of being watched, and if these computations are involved in driving social facilitation effects, then we would expect signals in dmPFC to be associated with individual-specific measures of social facilitation and concerns about how one is perceived by others. To test this hypothesis we calculated a measure of the extent of each participant’s social facilitation (i.e. the difference between observed and unobserved performance) and used this measure as a covariate in our group level analysis comparing social observation trials to non-observation trials that were successful. We focused on successful trials in this imaging analysis to ensure that there was a degree of independence between the behavioral covariate, which captured both successful and unsuccessful performance, and the fMRI analysis. We have utilized a similar approach in our previous studies of value-based motor performance (Chib *et al.*, 2012, 2014). We found that participants with greater activations in dmPFC, during trials in which their performance was observed compared to when it was not observed, exhibited greater amounts of social facilitation (Figure 2C [*x* = 9, *y* = 65, *z* = 10]; Figure 2D: average of all voxel within the independent ROI of dMPFC, *r* = 0.74, *P* < 0.001). Even at a very liberal threshold (*P* < 0.05 uncorrected) no other brain regions outside dmPFC were found to respond in this manner. We also performed ROI analyses of bilateral TPJ and found no significant correlations between activity in these regions and the social facilitation metric (rTPJ: *r* = 0.10, *P* = 0.68; lTPJ: *r* = 0.23, *P* = 0.35). Importantly, when adjusting for multiple comparisons across these regions, using a Bonferroni adjusted significance level of 0.017 (0.05/3), the dMPFC correlation still remains significant. These results align with previous studies of neural processing of audience observation (Somerville *et al.*, 2013; Müller-Pinzler *et al.*, 2015), and support the idea that activity in dmPFC plays a role in mediating social facilitation of performance.

To further test whether dmPFC activity is involved in tracking the effects of being watched, we also obtained an independent measure of the extent that participants are concerned about how they are perceived by others [using the Liebowitz Social Anxiety Scale (Liebowitz, 1987)], and related this social anxiety measure to participants’ parameter estimates extracted from voxels in dmPFC, when comparing the observed and unobserved conditions. Since social anxiety is related to the extent of one’s concern about being judged or evaluated by others, we expected that those participants who exhibited higher social anxiety scores would have greater dmPFC activation at the time of task performance. A linear regression of individuals’ social anxiety score and dmPFC activity, at the time of task execution, revealed a significant relationship (Figure 2E, *r* = 0.50, *P* = 0.03). A regression of individuals’ social anxiety scores and behavioral performance did not reach significance (*r* = 0.09, *P* = 0.71). While these results at least partially support our claim about the dmPFC’s role in social facilitation effects, it is important to interpret them with caution as the correlation with social anxiety measures is relatively weak, especially given that the sample size of our study is not optimized for detecting individual differences of this kind.

### Interaction between dmPFC and vmPFC during social facilitation

We tested the third prediction, that dmPFC and vmPFC interact as a function of social facilitation effects and task performance, by carrying out a PPI at the time of task execution. For this analysis we created another GLM in which activations in a seed region of dmPFC (identified in the aforementioned GLM analysis) were examined in relation to task-related whole brain functional connectivity. In particular, we were interested in how functional connectivity between dmPFC and other brain areas might change as a function of being observed during trials in which participants were successful. This analysis revealed that connectivity between dmPFC and vmPFC was increased on trials in which individuals were observed and their performance was successful or unsuccessful (Figure 2F and G;Table 3). Notably, we found that there was even more enhanced connectivity between these regions on unsuccessful observed trials, compared to successful observed trials. This affirms the link between these brain regions and illustrates a potential neural mechanism by which social computations about being observed could enhance performance reward outcome valuations. These results are consistent with previous studies of social decision-making that have reported enhanced functional connectivity between reward signals in vmPFC and mentalizing signals in dmPFC (Hampton *et al.*, 2008; De Martino *et al.*, 2013; Suzuki *et al.*, 2015).

**Table 3.** Connectivity analysis with dmPFC seed at the time of task execution as a function of social facilitation (Figure 2F) (*P *<0.005 uncorrected)

Region	Laterality	*x*	*y*	*z*	*T*-value
Ventromedial prefrontal cortex	C	6	53	−17	3.78
Premotor cortex	L	−12	−7	61	3.74
Premotor cortex	R	33	−4	55	4.33
Precuneus	C	−6	−52	46	3.80
Temporal parietal junction	R	−51	−46	10	3.99
Temporal parietal junction	L	51	−46	16	5.74

### Ventral striatum and social facilitation

**Fig. 3.**
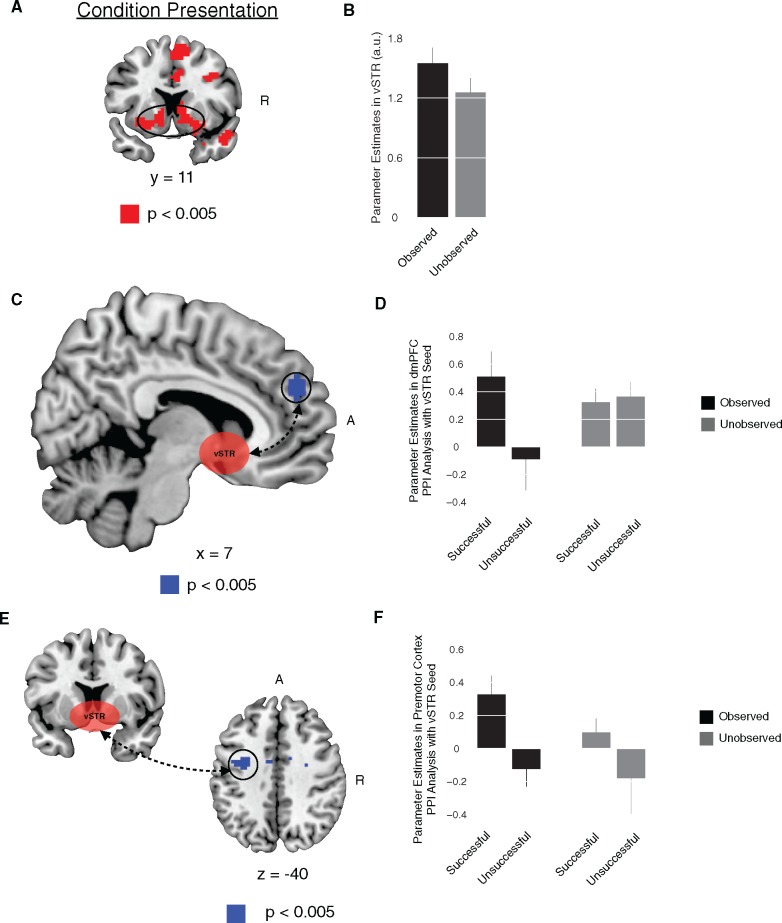
Ventral striatum and social facilitation. (A) At the time of condition presentation, vSTR exhibited significant increases in signal when comparing conditions in which participants’ performance was observed and unobserved. (B) Bar plot of the vSTR response for observed and unobserved conditions. (C) A connectivity analysis with vSTR seed, at the time of condition presentation, found that the functional coupling between vSTR and dmPFC was significantly increased during trials in which participants were observed and subsequently performed successfully. (D) Bar plot of the dmPFC PPI effects for the observed/unobserved conditions and successful/unsuccessful performance. (E) A connectivity analysis with a vSTR seed, at the time of task execution, found that the functional coupling between vSTR and premotor cortex was significantly increased during trials in which participants were observed and they were successful. (F) Bar plot of the premotor cortex PPI effects for the observed/unobserved conditions and successful/unsuccessful performance. All contrasts are significant at *P* < 0.05, small volume corrected. Error bars denote SEM. R, right. A, anterior.

We tested the fourth prediction, that the vSTR, which is a key structure involved in mediating motivation for action (Bray *et al.*, 2008; Talmi *et al.*, 2008; Balleine and O’Doherty, 2010), would show increased activity under observation, we examined brain activity at the time of initial condition presentation (when participants were first informed which type of condition they would be in—i.e. observed, unobserved). Our analyses were informed by previous studies, using the exact same motor task, that provided evidence consistent with a role for vSTR in mediating the influence of incentives on motor performance (Chib *et al.*, 2012, 2014). In the context of this study, we found enhanced activity in vSTR when comparing observed trials to unobserved trials, prior to task execution (Figure 3A and B, Table 4). Moreover, an analysis of functional connectivity between vSTR activity and dmPFC at the time of condition presentation, as a function of subsequent social facilitation, showed that the degree of coupling between these areas was correlated with behavioral effects of social facilitation within subjects (Figure 3C and D): on trials in which participants subsequently exhibited social facilitation they displayed enhanced functional connectivity between vSTR and dmPFC. No other brain regions were found to respond in this manner at a significance level of *P* < 0.05 uncorrected. Furthermore, examining whole brain functional connectivity at the time of task execution in a separate PPI analysis, with vSTR as a seed interacted with observation and performance conditions, revealed an enhanced coupling between vSTR and premotor cortex (Figure 3E and F;Table 5). In accordance with our previous findings (Chib *et al.*, 2012, 2014), these results are consistent with a role for vSTR underpinning motivational effects on motor performance. Our results are also consistent with the possibility that dmPFC inputs to vSTR may act on the motor drive that leads to social facilitation effects. However, it is important to note that our connectivity analyses do not allow direct inference about the directionality of the effects between dmPFC and vSTR and vSTR and premotor cortex.

**Table 4.** Regions with a significant increase in fMRI signal for observed *vs* unobserved conditions at the time of condition presentation (Figure 3A)

Region	Laterality	*x*	*y*	*z*	*T*-value
Ventral striatum	R	15	11	−8	4.16
Ventral striatum	L	−15	5	−11	4.26
Ventromedial prefrontal cortex	C	0	50	−17	4.68
Dorsomedial prefrontal cortex	C	9	59	16	4.98
Supplementary motor area	L	3	23	55	5.18
Precuneus	C	3	−52	28	5.28
Temporal parietal junction	R	63	−49	25	5.44
Temporal parietal junction	L	−36	−73	16	6.42

**Table 5.** Connectivity analysis with vSTR seed, at the task execution, as a function of social facilitation (Figure 3E) (*P *<0.005 uncorrected)

Region	Laterality	*x*	*y*	*z*	*T*-value
Premotor cortex	L	−33	−7	40	3.90
Somatosensory cortex	L	−9	−4	43	3.78
Somatosensory cortex	R	9	2	49	4.05

## Discussion

Our findings provide a neurobiological account of the social facilitation of monetarily incentivized behavioral performance. Brain regions previously found to be involved in reward outcome valuation, social cognition and motivation (Liljeholm and O’Doherty, 2012; Bartra *et al.*, 2013; Ruff and Fehr, 2014)—the vmPFC, dmPFC and vSTR—were found to exhibit increased activation during conditions in which participants’ performance is observed by an audience as compared to when they were not observed. Moreover, activity in dmPFC was related to the degree to which participants exhibited increased performance in response to social observation. Furthermore, the degree of coupling between dmPFC and vmPFC and dmPFC and vSTR was found to be related to such improvements in behavioral performance during social observation.

Our results suggest that during social facilitation, social signals represented in the dmPFC enhance reward value computations in vmPFC. Essentially, successful performance, when watched by others, increases reward representations in vmPFC. When examining the motivational signals related to social facilitation we found that vSTR had enhanced activity when individuals were observed compared to when they were not observed, and that social signals in dmPFC served to boost motivational signals in the vSTR. Moreover, these motivational signals in vSTR were coupled with premotor activity, suggesting a mechanism by which social motivation instantiated by dmPFC–vSTR connectivity influences motor execution. While our current experimental paradigm was not designed to examine the causal connectivity between brain activity in these regions, our results suggest that dmPFC encodes social signals which serve to enhance both value representations and motivated motor performance.

These results are consistent with an incentive-based account of social facilitation during skilled performance for monetary earnings—judgments of others modulate the reward-value associated with successful performance, and the motivation for subsequent performance. Our findings are consistent with a number of studies that have implicated mentalizing areas of the brain, in particular dmPFC, in encoding an individual’s sense of reputation during tasks in which on monetary incentives were not offered (Ochsner *et al.*, 2005; D’Argembeau *et al.*, 2007; Izuma, 2012; Somerville *et al.*, 2013; Müller-Pinzler *et al.*, 2015). Furthermore, our study extends this work by examining how social signals might serve to modulate reward outcome values and motivation during tasks in which individuals are watched by an audience and payment is contingent on performance.

Our findings also have implications for psychological explanations of audience effects and social facilitation. According to our incentive-based account being watched by others serves to increase motivation and outcome values, and these changes could result in allocation of attentional resources away from the skilled-motor task. In this sense divergence of attention resulting from concern about the audience may provide a potential role in modulating facilitation. However, we did not find evidence for behavioral measures correlating with the front-parietal attention network (Corbetta and Shulman, 2002), as would be predicted by attentional theories. While this does not completely rule out attention in the phenomenon, such effects do not appear to be mediated by the neural networks commonly implicated in controlling attention. Instead, our findings of increased dmPFC activation during social facilitation, and correlations between social anxiety scores and behavioral effects, align more with a self-presentation account of social facilitation in which the presence of an audience makes participants concerned about how they are perceived by others (Bond, 1982; Bond and Titus, 1983). These social concerns may then lead to increased drive and facilitation effects on performance.

Our investigation focused on the brain activity responsible for encoding the cognitive states related to social facilitation, and we were especially careful to control for motoric performance confounds during our experimental analyses. Our analysis of differences in neural activity between observed and unobserved trials revealed greater activity in the vSTR, at trial onset. This is in keeping with other experiments showing that vSTR encodes motivational signals related to anticipation of future rewards during instrumental tasks (Knutson *et al.*, 2001; Pessiglione *et al.*, 2007; Talmi *et al.*, 2008; Chib *et al.*, 2012), and facilitation of behavioral performance in the context of negative evaluation (Prevost *et al.*, 2017). Our functional connectivity analysis illustrated an increase in connectivity between vSTR and premotor cortex on trials in which social facilitation occurred, suggesting that the vSTR might serve as the motivational interface that drives motoric performance resulting in social facilitation.

Previous studies have provided evidence that activity in brain systems involved in neuroeconomic valuation exhibit task-relevant coupling with areas involved in social cognition such as dmPFC (Hampton *et al.*, 2008; De Martino *et al.*, 2013; Suzuki *et al.*, 2015). For example, these studies have found that representations of the value of particular decision options within the valuation system are modulated by social computations involving: the strategy being adopted by other agents (Hampton *et al.*, 2008); beliefs about group behavior (Suzuki *et al.*, 2015); or even the collective behavior of financial markets (De Martino *et al.*, 2013). One limitation of this study is that the nature of the phenomenon being studied is such that we could not obtain insight into the specific nature of the social computations being implemented within the social cognition network that act to influence brain systems involved in valuation and motivation, as the current task paradigm is not amenable to a computational model-based approach. Future studies could begin to probe the nature of the computations by for example deploying multivariate analysis tools to determine whether other agents in the audience are specifically encoded at particular times during the task, and if those representations are modulated by successful *vs* unsuccessful performance. Another important future direction would be to begin to unpack the temporal dynamics of audience effects on behavior, perhaps using techniques with more finegrained temporal resolution than fMRI such as MEG or EEG, as this might enable more robust inferences about the directionality of the interaction effects between the different systems that underpin the behavioral effects of social facilitation.

It is also important to note that a number of studies have reported audience effects that lead to *decreases* in performance or ‘choking’ under pressure (Bond and Titus, 1983), yet here we examined the case in which social situations lead to increases in performance. It is possible that social inhibition effects of performance could occur under conditions in which larger audiences are present, when large incentives are offered while being viewed by an audience, or as the result of an interaction between incentives and social cues. It seems plausible that a similar network of brain regions as those mediating social facilitation may also be involved in such social choking effects. Notably, while we found overall increased connectivity between dmPFC and vmPFC when comparing observed to unobserved trials, we found that trials that were unsuccessful yielded the greatest degree of coupling between these regions. This suggests the possibility that a similar mechanism might underlie effects of observation on deleterious effects on performance, although such behavioral effects were not found in this study. Investigating the role of the regions we have identified here during other aspects of social observation and performance will be an important future direction in dissecting the general neural signals that influence both facilitory and deleterious influences of observation on performance.

An improved understanding of the neurobiology of audience effects on performance will have applications in a myriad of environments in which individuals’ performance is observed by others, such as employees being observed their managers, students being evaluated by their teachers, and even patients being monitored by their physical therapists. By understanding how such social situations influence performance, we may design better social environments that can enhance behavioral performance and learning.

## Funding

This study was funded by grant NSF 1062703 from the National Science Foundation to J.P.O.D. V.S.C. was supported by the Eunice Kennedy Shriver National Institute Of Child Health & Human Development of the National Institutes of Health under Award Number K12HD073945. R.A. was supported by a fellowship from the Nakajima Foundation.

*Conflict of interest.* None declared.
